# Book Review: Els jocs i els esports Tradicionals. Tradicionari. [The Traditional Games and Sports. Traditionari]

**DOI:** 10.3389/fpsyg.2020.605920

**Published:** 2020-11-16

**Authors:** Biel Pubill-Soler

**Affiliations:** Institut de Flix, Departament d'Educació, Generalitat de Catalonia, Flix, Spain

**Keywords:** intangible cultural heritage, encyclopedy, communities, transdisciplinarity, cultural diversity, motor action science, ethnomotricity

Between 2005 and 2008 the accredited publishing house Enciclopèdia Catalana published *Tradicionari. Enciclopèdia de la cultura popular de Catalunya* (see Figure 1), a huge work of 10 volumes. The aim of this ambitious Encyclopedia was to show the different cultural dimensions of the Catalan popular tradition. The domains of the work, the party and the festive calendar, music and dance, myths, beliefs, and traditional games and sports are some of the themes of the encyclopedia.

**Figure 1:**
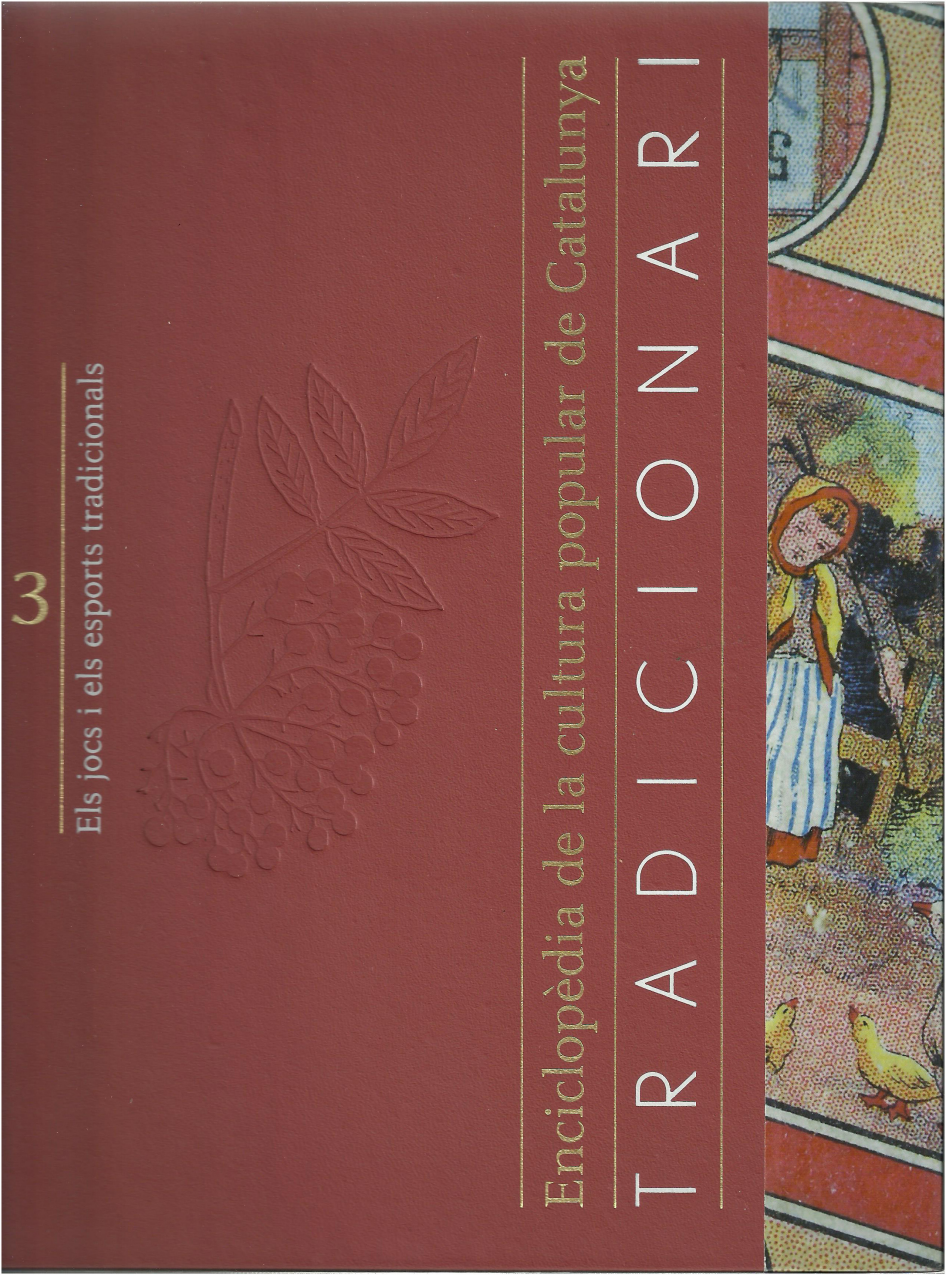
Cover of the book Els jocs i els esports Tradicionals. Tradicionari. [The Traditional Games and Sports. Traditionari].

The contend of each volume is based on studies of a wide crowd of specialists: Eminent folklorists, ethnographers, and anthropologists of intangible cultural heritage. The challenge is to join the tradition and modernity in a whole approach, in order to understand the contribution of these manifestations in the past, in the present and probably in the future local societies.

Although the *Tradicionari*. is a collective work, it is presented as a joint, integrated and coherent work. This challenge was achieved thanks to Joan Soler-Amigó, the director of the 10 volumes, surrounded by the best specialists in each field, becoming the coordinators of each volume.

The third volume of this encyclopedy is dedicated to traditional games and sports (TSG). A team of more than 25 specialists, coordinated by Pere Lavega-Burgués -professor at INEFC, University of Lleida, Spain, current president of the European Association of Games and Traditional Sports. He is also expert of UNESCO and author of an extensive bibliography in the field of physical activity and TSG. This team guarantees a broad and transversal vision, with multidisciplinary approaches and a range of perspectives that between theory and practical exemplification give the right balance to the work.

The scientific rigor is already evident in the first pages with Prof. Pierre Parlebas' contribution who succinctly, intelligently and understandably writes about the game as an inherent part of the culture, and eloquently introduces some of the basic concepts of praxeological study.

Parlebas, creator of the science of motor action or motor praxeology, establishes at the outset some knowledge and an original approach in relation to the internal logic of the games (relations between the players, space, materials and time of game). He also explains the connection with other sociocultural factors of the local culture (external logic). Both dimensions internal and external logic are integrated in the concept of *ethnomotricity* to understand the meaning of any TSG in its society.

Pere Lavega, coordinator of this volume, takes over by defining the concept of the game, exposing different classical theories related to the game to end up delving into the praxeological analysis, with a variety of traditional games and sports.

These first chapters are key pages to understanding how this volume of the Traditional is structured. From the exposition of the scientific theories on the game we find that four of the nine sections that follow take the name, precisely, of the determining aspects that conform the internal logic of the games as well the external logic: Protagonists of the games, Toys—materials of game—, Game Spaces and Game Times.

The encyclopedia develops the chapters in a original and very rigorous way. It not only describes a great repertoire of TSG in Catalonia, but also presents this playful universe joined to the past and also to the present. The volume notes the evolution and changes undergone while pointing and predicting future trends. Thus, for example, when dealing with toys—play materials—the importance of handcrafted construction is emphasized. Then, firstly, the world of collecting and the role of museums is described, and secondly the visibility is also given to modern facilities such as toy libraries or new trends like videogames in the current digital society.

In the same way, the section The Spaces of the Games is not limited to enumerate traditional spaces of game as for instance, the house, the streets and places or near natural surroundings. The section also deals with the domestication of spaces—parks, gardens, sports facilities…—or the creation, consolidation, and development of theme parks. And of course, as a current and future trend, the emergence of a new play space stands out: The internet.

Another quality to note is that from the first to the last page, the text is enriched by many examples of TSG related to each section. To complete this approach, the last two chapters are reserved to present an extensive and significant thematic repertoire of TSG taking into account criteria as: TSG to start playing; TSG with different sort of relationships: individual challenges (e.g., skittles, races, jumping games) cooperation (dances and rhythmic games), opposition (e.g., wrestling and tag games), team games (e.g., ball games, dodgeball); winning or losing TSG (e.g., competitive TSG); and also board games.

*Traditionari* becomes a new encyclopedic concept that, without renouncing the enumeration or collection of TSG, this book seeks to be a reference for consultation as well as a source of pedagogical and creative inspiration. The readers can take profit of a whole section dedicated to explain the socialization role of TSG as well as many examples that TSG offers as a pedagogical tool in the field of education and recreation. This section also presents some evidences to identify the effects of TSG on emotional well-being, as a tool for mediation and resolution of conflicts.

The book provides an excellent approach to past (tradition) and present (modernity) in order to understand that TSG foster important values for current society. *Traditionari* is the result of a rigorous research of the Catalan TSG as Intangible Cultural Heritage and many unpublished contributions of prestigious specialist. And all of this, accompanied by numerous examples of good practices.

Another detail should be highlighted as a last remark; this volume presents a large number of illustrations related to old and current photographs carefully chosen. The result is an attractive and stimulating strategy to provide the core messages. The book invites the reader to understand the contribution of ludic intangible cultural heritage in ancient ages, in current society and also to think about its contribution in the future.

Finally, we the book has as limitation that the content is written in Catalan, although on the other hand we see that it is available online.

## Author Contributions

The author confirms being the sole contributor of this work and has approved it for publication.

## Conflict of Interest

The author declares that the research was conducted in the absence of any commercial or financial relationships that could be construed as a potential conflict of interest.

